# Identification and characterization of novel antimicrobial peptides from *Camelus dromedarius*: a combined bioinformatics and experimental study

**DOI:** 10.3389/fimmu.2026.1745714

**Published:** 2026-01-22

**Authors:** Wafa Al-Mamari, Yasmin Elhag, Samir Al Bulushi, Rokeya S. Rekha, Cecilia Mörman, Peter Bergman, Aliya Al-Ansari, Salma Al-Adwani

**Affiliations:** 1 Department of Biology, College of Science, Sultan Qaboos University, Muscat, Oman; 2 Department of Animal & Veterinary Sciences, College of Agricultural and Marine Sciences, Sultan Qaboos University, Muscat, Oman; 3 Animal Research Center, Directorate General of Veterinary Services, Royal Court Affairs, Muscat, Oman; 4 Department of Laboratory Medicine, Division of Clinical Immunology, Karolinska Institutet, Stockholm, Sweden; 5 Department of Clinical Immunology and Transfusion Medicine, Karolinska University Hospital, Stockholm, Sweden; 6 Department of Medicine, Karolinska Institutet, Huddinge, Sweden; 7 Center for Life Sciences, Paul Scherrer Institute, Villigen, Switzerland

**Keywords:** antimicrobial peptide, bioinformatics, *Camelus dromedarius*, cathelicidin, multidrug resistance

## Abstract

There is an urgent need for new antimicrobial agents to address the emerging antimicrobial resistance and the lack of novel antibiotics on the market. Antimicrobial peptides (AMPs) have gained significant interest as potential antibiotics over the past 30 years due to their broad activity against bacteria. So far, the presence, characteristics, and function of AMPs in camel immunity remain to be explored. Therefore, this study aims to identify and functionally characterize AMPs in *Camelus Dromedarius* using *in-silico* and experimental approaches. *In-silico* identification and prediction of cathelicidin peptides properties were conducted using Blastp, Conserved Domain, Signal P-5.0, Peptide Cutter-Expasy, and the Antimicrobial Sequence Scanning System (AMPA) database. Physicochemical and biological properties were characterized using bioinformatics analysis tools. The experimental assays of synthetic AMPs were performed using circular dichroism (CD) spectroscopy, colony-forming assay, sytox green uptake assay, transmission and scanning electron microscopy, and hemolysis assay. Three cathelicidin peptides were identified from *Camelus Dromedarius* which were designated as CdPMAP-23, Cdprotegrin-3 (CdPG-3), and Cdcathelin-like (CdCATH). CdPG-3 and CdCATH demonstrated significant antibacterial effects against all tested Gram-negative and Gram-positive strains, including *Escherichia. coli* (Multidrug resistant) and *Methicillin-Resistant Staphylococcus aureus* (ATCC 700699). These two peptides caused significant membrane leakage and damage to *Escherichia. coli* (ATCC 25922), with CdPMAP-23 showing a lesser effect. Lower concentrations of CdPMAP-23, CdPG-3 and CdCATH exhibited low to moderate lytic activity against red blood cells in humans, camels, and chickens. This study identified novel AMPs from dromedary camels with potential therapeutic value against multidrug-resistant strains. The results show that AMPs are present in dromedary camels, setting out a foundation for further studies on the unique features of their innate immune system.

## Graphical Abstract

**Figure:**
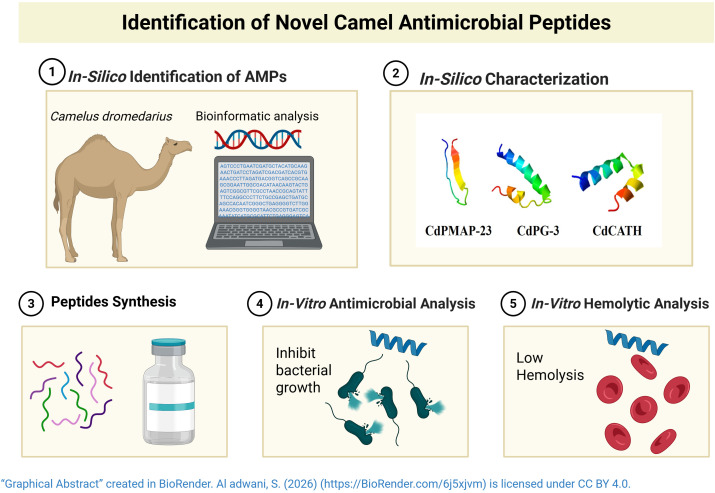

## 1 Introduction

Antibiotics are effective in treating pathogenic bacteria (1). Using antibiotics in large quantities or even excessive use gradually led to the emergence of bacteria resistant to antibiotics (2) posing a serious challenge to human health (3, 4). Most conventional antibiotics affect bacterial metabolism and proliferation by inhibiting or killing bacteria through interacting with targets via site-specific binding mechanisms (5–7). As a result, this mechanism of site-specific interaction is also susceptible to increasing the chance of antibiotic resistance, as some mutations in the binding sites or modifications to the antibiotics structure could result in antibiotic deactivation (8). There has been a notable reduction in the exploration and creation of new categories of antibiotics. Consequently, in order to combat the widespread resistance to antibiotics, new potent antimicrobials are urgently needed that act through alternative mechanisms (9, 10).

Over the last 30 years, antimicrobial peptides (AMPs) have received substantial attention as potential alternatives to antibiotics (3, 11). AMPs are a diverse group of small bioactive proteins that are part of the body’s innate defense mechanisms against pathogens (12). AMPs have great potential as antimicrobials due to bacteria’s limited ability to develop resistance (13). Defensins and cathelicidins are the two primary AMPs in mammals. They are small, cationic, and amphipathic molecules mainly killing bacteria by making pores in the cell wall. Furthermore, these peptides have been shown to exhibit chemotactic properties and promote wound healing, as well as modulate the immune response (14). Mammalian cathelicidins were initially identified in the neutrophils of bovine and are known to have a broad spectrum of bactericidal activities (15). Cathelicidins have a highly conserved signal peptide at the N-terminus, a pro-domain (cathelin domain), and a heterogeneous antimicrobial domain at their C-terminus (12). Cathelicidins peptides are found in mast cells, macrophages, neutrophils, and epithelial cells (16). Moreover, cathelicidins play a crucial role in specific defense mechanisms found in epithelial cells in the oral mucosa, lungs, skin, gut, testis, and urinary bladder and can prevent the growth of bacteria (17, 18).

Dromedary camels (*Camelus dromedaries*) are economically and culturally significant in the Sultanate of Oman and across the Mediterranean region, valued for their meat, milk, and utility in racing competitions. Interestingly, camels are less susceptible to several infectious diseases (19), such as foot and mouth disease (FMD, tetanus (bacteria), and bovine spongiform encephalopathy (prion disease), when compared with sheep living in similar conditions (20, 21). Recently, the number of researches on understanding the camel immune system has been increasing since the discovery of their unique single-domain antibodies (22, 23). Recent research in Oman confirmed that camels have a strong innate and adaptive immune system. They can respond better to bacterial and viral infections compared to other domesticated animals. This ability makes them less likely to get certain illnesses that commonly affect other ruminants (24).

Despite advancements in camel immunology, there remains a lack of in-depth knowledge on the presence of AMPs and their detailed characteristics and functions in protecting against pathogens, as well as their role in immunomodulatory functions. *In-silico* methods have facilitated the design of highly effective antimicrobial, antiviral, antifungal, anticancer, and immunomodulatory peptides (25). Recently, more researchers are focusing on investigating computational approaches that utilize physicochemical and structural features to develop effective and diverse peptides (26). AMPFinder is one example of the computational models that have been recently developed to identify the AMPs and their roles based on peptide sequence information (27).The proteins and peptides found in camel milk, including lactoferrin, lactoperoxidase, and lysozyme, significantly contribute to the milk’s overall antimicrobial effectiveness. These peptides can serve as natural alternatives to antibiotics because of their antibacterial characteristics (28). One study isolated crude protein from camel leukocytes and found that they have antibacterial activity against some multidrug-resistant pathogens (29). Another study characterized the Arabian camel hepcidin nucleotide sequence (β-defensin-like peptide) using bioinformatic analysis (30). Therefore, the aim of this study is to predict novel AMPs from dromedary camels using an *in-silico* approach and to further characterize them experimentally, potentially utilizing them as therapeutic drugs (antibiotics).

## 2 Materials and methods

### 2.1 Computational analysis

#### 2.1.1 Identification of cathelicidin peptides

Experimental and *in-silico* approaches were used to identify and characterize cathelicidin peptides from dromedary camels. The experimental LC-MS/MS results suggested that camel crude leukocyte-derived polypeptides likely contain cathelicidin-like peptides that matched 86% of Prophenin-2 of Sus scrofa (P51525) (**Supplementary Material 1**). Subsequently, bioinformatic analyses were performed to identify multiple potential antimicrobial peptides in the camel genome. The accession numbers and sequences of known cathelicidin peptides from other *Camelidae* family species, including CATH-6 from *Camelus ferus* and cathelicidin from *Camelus bactrianus* and VpCATH-22, VpCATH-24, VpCATH-26, VpCATH-29, VpCATH-35, VpCATH-38, Vp-prophenin, and VicBac from the alpaca *Vicugna pacos* (31) were retrieved in FASTA format. To identify the best-matching sequences for annotated cathelicidin peptides in camels, BlastP at the NCBI database was used, restricting the search by taxonomy to *Camelus dromedarius* (9838). Then, the identified potential proteins were analyzed using SignalP 5.0 to predict the signal peptide and propeptide regions (https://services.healthtech.dtu.dk/services/SignalP-5.0/). The peptide cutter-Expasy tool was used to predict potential cleavage sites cleaved by the neutrophil elastase enzyme (https://web.expasy.org/peptide_cutter/). The Antimicrobial Sequence Scanning System (AMPA) database was utilized to detect antimicrobial active regions with a threshold value of 0.225 (https://tcoffee.crg.eu/apps/ampa/do).

#### 2.1.2 Comparison of identified camel cathelicidin peptides with cathelicidins from other species

To compare the full protein sequences of identified peptides with known mammalian cathelicidins from different species, multiple sequence alignment was performed using Uniprot alignment, and also each identified mature peptide was aligned with known cathelicidin peptides of other *Camelidae* family species (https://www.uniprot.org/align).

#### 2.1.3 Physicochemical, helical and 3D structure properties

The physicochemical characteristics of the identified peptides, such as peptide length, net charge, hydrophobic ratio, molecular weight, and isoelectric point, were assessed using the Compute pI/MW tool from Expasy (https://web.expasy.org/compute_pi/) and the Antimicrobial Peptide Database Calculator Predictor (APD3) (https://aps.unmc.edu/prediction). The α-helical and 3D structure of the three identified peptides were predicted by the HeliQuest database (https://heliquest.ipmc.cnrs.fr/cgi-bin/ComputParamsV2.py) and the I-TASSER server (https://zhanggroup.org/I-TASSER/). I-TASSER predicts protein structures through four main steps: identifying threading templates, assembling structural fragments, refining models, and annotating protein functions based on their structure (32).

#### 2.1.4 Assessment of the membrane-binding ability of identified peptides

The Boman index demonstrated the capacity to adhere to the cell membranes or various proteins, which is calculated as the total of the amino acid solubility values within a peptide sequence (33). It was assessed using APD3 (https://aps.unmc.edu/prediction). The TMHMM web server (https://services.healthtech.dtu.dk/services/TMHMM-2.0/) was used to evaluate the cellular localization of the identified peptides and their potential to attach to the negatively charged bacterial cell membranes.

#### 2.1.5 Prediction of biological properties

The CAMP_R4_ database was operated to predict antimicrobial sequences using an algorithm developed for natural and synthetic AMP. To estimate the antimicrobial potential of the identified peptides, three machine-learning algorithms in the CAMP_R4_ database were used for natural and synthetic peptides: Support Vector Machine (SVM), Artificial Neural Network (ANN), and Random Forest (RF) (https://camp.bicnirrh.res.in/predict/). A peptide with a higher probability indicates a greater likelihood of being antimicrobial; probabilities range from 0 to 1. It is important to note that the software intrinsically determines the threshold and cannot be modified by the user. The web server AVPpred was used to predict the antiviral action (http://crdd.osdd.net/servers/avppred/submit.php). It involves four distinct models: First, the AVP motif, which yields YES or NO; second, the Alignment model, which yields AVP or Non-AVP; third, the Composition model; and fourth, the Physico-chemical model, which produces numerical data (in the form of percentages). If the peptide data showed a potential antiviral action, the overall result is indicated with a YES; if not, it is represented with a NO. Antifungal activity was predicted using the Antifp server, which returns the result as a number score. A threshold of 0.5 was applied for this investigation.

#### 2.1.6 Hemolysis prediction

The Hemolytic Peptide Identification Server (HemoPI) was utilized to estimate the hemolytic activity of identified peptides. It allows for the screening of hemolytic or non-hemolytic peptides ranked by their probability of being hemolytic, ranging from 0 to 1 (1 is very likely to be hemolytic, while zero is very unlikely to be hemolytic) (https://webs.iiitd.edu.in/raghava/hemopi/).

### 2.2 Peptide synthesis

In this research, the identified peptides, along with the reference peptide (LL-37) were produced using solid-phase synthesis by GL Biochem in Shanghai, China, with fluorenyl methoxycarbonyl (Fmoc). The purity of each peptide was examined using reverse-phase high-performance liquid chromatography (RP-HPLC) and MALDI-TOF mass spectrometry, confirming that each peptide had a purity greater than 98%. For all experiments, the peptides were solubilized in 0.1% trifluoroacetic acid (TFA) in water and stored at -80 °C for future use.

### 2.3 Experimental analysis

#### 2.3.1 Circular dichroism spectroscopy

The circular dichroism (CD) spectra for the three peptides (CdPMAP-23, CdPG-3, CdCATH) were obtained at a concentration of 30 µM using a Chirascan Circular dichroism spectrophotometer (Applied Photophysics, U.K.), fitted with a temperature-controlled Peltier system to monitor the peptides’ secondary conformations under varying experimental conditions. Quiescent conditions within a 190–260 nm spectral range, 1 nm of bandwidth and step size/resolution, and a 2-second interval for each point were used. A 2 mm path length quartz cuvette was utilized for the measurements, and all measurements were performed at RT. Buffer background spectra were removed, and results were expressed in terms of mean residue molar ellipticity. The addition of SDS to an ending concentration of 0.2% and the titration steps of LPS were performed in 10 mM sodium phosphate buffer at pH 7.4. The α-helical content was calculated using Eq. 1 (34):


$$\alpha -helical\;content\;\left[\%\right]=\;\left(\frac{\theta_{222\;nm,\;random\;coil}-\theta_{222\;nm,\;\;observed}}{\theta_{222\;nm,\;random\;coil}-\theta_{222\;nm,\;\;\alpha -helix}}\right)*100,$$

where the average ellipticity for random coil structures is 3.900 deg cm^2^ dmol^−1^ and the mean of the ellipticity for α-helices is −35.700 deg cm^2^ dmol^−1^.

#### 2.3.2 Bacterial culture

Bacterial cultures, including *Staphylococcus. aureus* (ATCC 25923), *Methicillin-Resistant Staphylococcus aureus* (ATCC 700699), *Escherichia. coli* (ATCC 25922), *Escherichia. coli* (MDR), *Klebsiella pneumonia* (ATCC 1705), and *Klebsiella pneumonia* (ATCC 1706) were obtained from the Department of Microbiology and Immunology, College of Medicine and Health Science, Sultan Qaboos University. They serve as standard reference strains for measuring antimicrobial susceptibility, studying biofilm formation, and performing multiple microbiological analyses. The antibiotic susceptibility test was performed for each bacterial strain using the Automated Identification and Susceptibility Testing System AF-300 (Mindray Company, China) (**Supplementary Tables 1**-**6**).

#### 2.3.3 Colony forming assay

The antibacterial activity of synthesized peptides was evaluated using a colony-forming assay. Each bacterium was grown in Luria-Bertani (LB) broth (Hardy Diagnostics, Santa Maria, USA) to mid-logarithmic phase for two hours, and the optical density was adjusted to 0.1 subsequently. Then, each bacterium was treated with a range of peptide concentrations (from 1.25 µM up to 160 µM) for 3 h at 37°C, shaking. After conducting serial dilutions, the samples were spotted (25 μL) onto LB agar plates. The plates were incubated overnight at 37°C, after which the colonies of surviving bacteria were enumerated.

#### 2.3.4 Sytox green uptake assay

The approach employed to assess the permeabilization of the inner membrane of cells was derived from a previous study (35). *E. coli* ATCC 25922 was cultured in LB broth (Hardy Diagnostics, Santa Maria, USA) until it reached the mid-logarithmic growth phase, which was achieved in approximately two hours. A concentration of 5 × 10^7^ CFU/mL of the cultured *E. coli* was subsequently transferred into a black 96-well plate and exposed to varying concentrations of synthetic peptides ranging from 1.25 µM to 160 µM for 30 minutes at 37°C. Post-incubation, the bacterial cultures were centrifuged at 1,200 × g for 10 minutes at RT and then washed once with PBS. The resulting bacterial pellet was resuspended in 100 µL of 3 μM SYTOX Green Nucleic Acid Stain (Invitrogen, Thermo Fisher Scientific, US) and incubated for an additional 5 minutes at RT. Finally, fluorescence intensity was measured at an excitation wavelength (λ_ex_) of 504 nm and an emission wavelength (λ_em_) of 523 nm using a Varioskan LUX Multimode Microplate Reader (Thermo Scientific).

#### 2.3.5 Transmission and scanning electron microscopy

Transmission electron microscopy (TEM) was employed to investigate the effects of CdPMAP-23, CdPG-3, and CdCATH on the cellular structure of *E. coli* ATCC 25922. In parallel, scanning electron microscopy (SEM) was performed to examine any modifications to the bacterial membrane. These methodologies for TEM and SEM were implemented in accordance with established protocols from prior research (36). *E. coli* was cultured to the logarithmic phase and subsequently diluted to a concentration of 5 × 10^8^ CFU/mL in LB broth. The culture was divided into two groups: one served as a negative control (untreated), while the other was subjected to treatment with 80 µM of each peptide at 37°C for 30 minutes. For TEM preparation, the treated mixtures were fixed with 2.5% glutaraldehyde in 0.1 M PBS (pH 7.4) for 30 minutes, followed by washes with PBS. Post-fixation was conducted using 2% osmium tetroxide in PBS for 2 hours at 4°C. The samples were then dehydrated sequentially in ethanol and acetone before being embedded in LX-112 resin. Ultrathin sections were prepared using a Leica EM UC7 ultramicrotome (Leica Microsystems) and subsequently stained with uranyl acetate and Reynolds’ lead citrate. The resulting TEM sections were imaged using a Tecnai Spirit G2 Bio TWIN Electron microscope (Tecnai High Technologies), operating at 100 kV, with image capture performed using a 2k × 2k Veleta CCD camera (Olympus Soft Imaging Solutions GmbH, Münster, Germany). To prepare samples for SEM, 2.5% glutaraldehyde PBS was used to fix them. After adhering fixed samples onto a pore membrane, they were rinsed thoroughly with Milli-Q water. Dehydration was achieved through a graded ethanol series, followed by critical point drying utilizing CO2 (Leica EM CPD 030). Carbon adhesive tabs were applied, and the pore membranes were then sputter-coated with platinum (Quorum Q150T ES) to secure them onto specimen stubs. SEM imaging was conducted using an Ultra 55 field-emission scanning electron microscope (Zeiss, Oberkochen, Germany) at an accelerating voltage of 5 kV, with images captured via the SE2 detector. TEM and SEM analyses were repeated in triplicate.

#### 2.3.6 Hemolysis assay

Human, camel, goat, and chicken blood were collected in Heparin-coated tubes. Ethical approval was obtained from Sultan Qaboos University for the use of camel blood (SQU/EC-AUR/2023-2024/5), goat blood (SQU/EC-AUR/2023-2024/6), human blood was obtained from the blood bank at SQUH, and chicken blood (SQU/EC-AUR/2024-2025/35). The blood samples of camels were collected from the Royal Camel Corps (RCC), while the goat and chicken blood samples were obtained from the Sultan Qaboos University farm (SQU). The hemolysis assay was performed based on an earlier study (35). Whole blood was subjected to centrifugation at 800 × g for 10 minutes at RT to separate the erythrocytes from the plasma. Following plasma removal, erythrocytes were thoroughly rinsed three times with PBS and subsequently resuspended in a 1% solution of PBS (vol./vol.). The resuspended erythrocytes were then plated in a microplate of U-bottom wells and mixed with varying concentrations of peptides (1.25 to 160 µM). The incubation was conducted for one hour at 37°C under continuous agitation. Post-incubation, the plates were centrifuged again at 800 × g for 10 minutes to pellet the erythrocytes. Following this, 80 µL of the supernatant from each well was transferred to a clear 96-well plate. The absorbance of the released hemoglobin was quantified at 540 nm using a Multiskan GO microplate reader (Thermo Scientific). The hemolysis percentage was determined according to the following formula: (OD of treatment-OD of negative control)/(OD positive control-OD of negative control) ×100) where the negative control (without peptides), was considered as 0% lysis, while a positive control (1% Triton X-100), was considered as 100% lysis. The hemolysis percentage was determined, and data normalization was performed using the average from three experimental repetitions. A 1% solution of Triton X-100 served as the positive control, whereas PBS was used as the negative control. According to biological evaluation of medical devices (ISO 10993-4:2017), the guidelines for Hemolysis are classified as follows: % of hemolysis ≤ 2% is non-hemolytic, % of hemolysis is between 2–5% is slight hemolysis, % of hemolysis between 5–10% is moderate hemolysis, and % of hemolysis > 10% is severe hemolysis. A study shows that % of hemolysis ≤ 5% considered safe for clinical use, and % of hemolysis >10% is unsafe (37). Additionally, research indicates that ≤ 5% of hemolysis is a golden standard for peptide safety (38).

#### 2.3.7 Statistical analysis

A Microsoft Excel spreadsheet was used to export the results. Statistical analysis was performed using GraphPad Prism version 10.4.1 (GraphPad, La Jolla, CA), which included log-transformed and one-way ANOVA for CFU count data. A p-value of ≤ 0.05 was considered statistically significant. Experiments were done in triplicate and were presented as mean ± SEM.

## 3 Results

### 3.1 Computational analysis

#### 3.1.1 Identification of cathelicidin AMPs from dromedary camels

**Figure 1:**
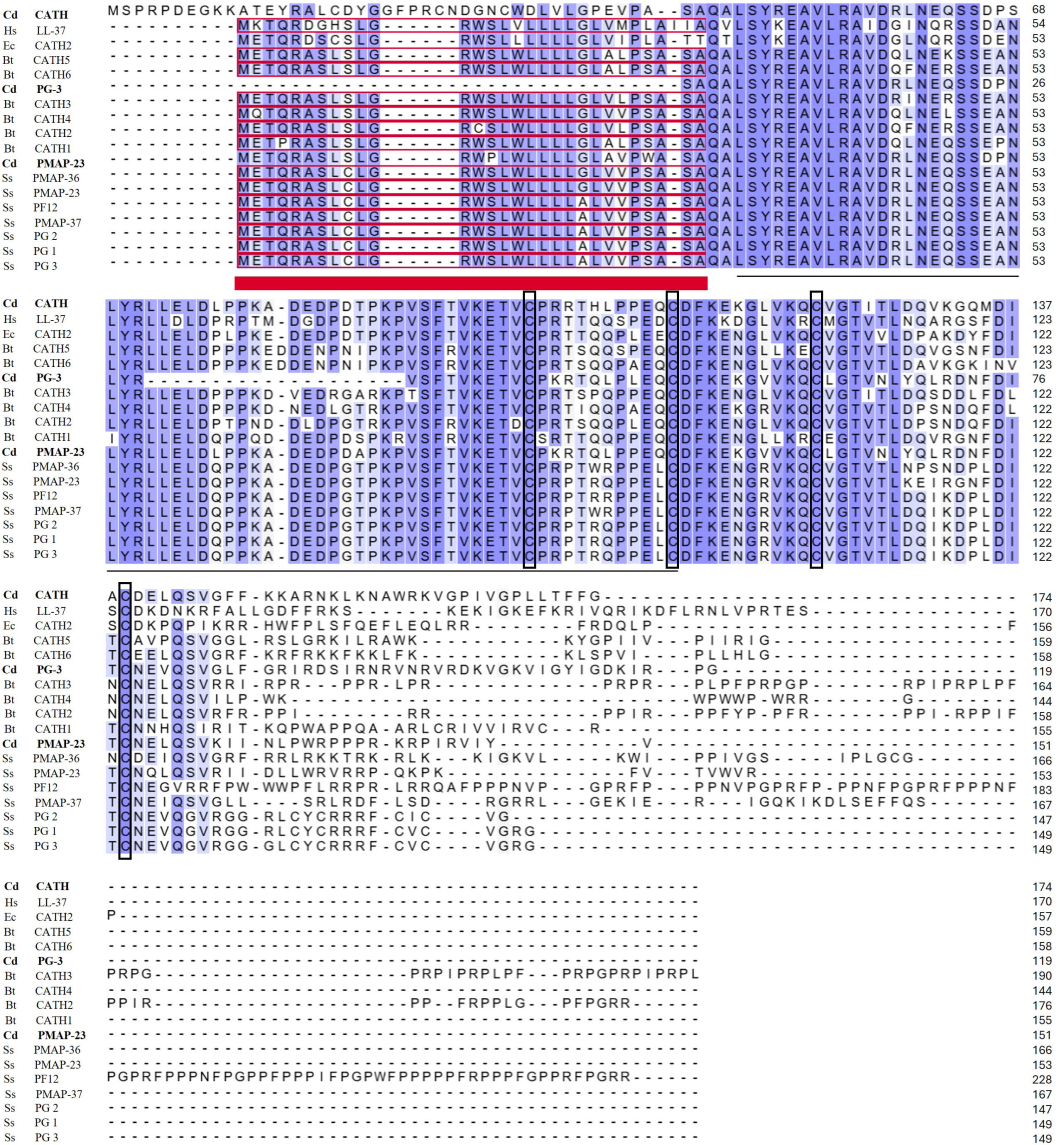
Full protein sequence alignment of identified cathelicidin peptides with homologs from other species. Full-length amino acid sequences of camel cathelicidins were aligned with cathelicidins from human, equine, bovine, and porcine species to highlight conserved structural features using Uniprot alignment. The signal peptides are underlined in red, while the cathelicidins’ conserved domain is underlined in black. The conserved cysteine residues are framed. The novel camel peptides (CdCATH, CdPG-3, and CdPMAP-23) are bold. The accession numbers of related sequences are: human (Hs) LL-37, P49913; equine (Ec) CATHL2, O62841; bovine (Bt) CATHL5, P54229; Bt CATHL6, P54228; Bt CATHL3, P19661; Bt CATHL4, P33046; Bt CATHL2, P19660; Bt CATHL1, P19660; Porcine (Ss) PMAP-36, P49931; Ss PMAP-23, P49930; Ss PF12, P51525; Ss PMAP-37, P49932; Ss PG-2, P32195; Ss PG-1, P32194; Ss PG-3, P32196.

According to the cathelicidin conserved domain, the best hits from *Camelus dromedarius* were CdPMAP-23, Cd protegrin-3 (CdPG-3), and Cd cathelin-like (CdCATH) with accession numbers XP_031326097.1, KAB1264031.1, and XP_031326999.1, respectively. All identified cathelicidin peptides were predicted using BlastP at NCBI. The prediction of the presence of signal peptides in sequences using Signal P-5.0 showed that CdPMAP-23 was highly likely to be secreted outside cells. The prediction of cleavage sites using the peptide cutter-Expasy tool demonstrated that the enzyme would cut at the first valine residues in exon4 in all identified peptides, including LL-37 (reference peptide). In addition, all peptides were predicted to have antimicrobial activity based on the Antimicrobial Sequence Scanning System (AMPA) database (**Table 1**). The protein sequences of CdPMAP-23, CdPG-3, and CdCATH were aligned with known mammalian cathelicidins from different species, including equine (Ec), bovine (Bt), human (Hs), and porcine (Ss), and showed a highly conserved pro-region at the N-terminal cathelin-like domain (CLD) with four cysteines (**Figure 1**). In contrast, the mature antimicrobial peptides in the C-terminal region exhibited considerable variability in length and sequence. The identity matrix indicated that CdPMAP-23 displayed 72-74% identity with SsPMAP-23, and CdPG-3 showed 62-66% identity with SsPG1, SsPG2, and SsPG3. Meanwhile, CdCATH exhibited a lower identity, ranging from 54% to 65%, when compared to cathelicidins from human, porcine, and bovine species. Therefore, each identified mature peptide was aligned with known cathelicidin peptides of other Camelidae family species, including *Camelus ferus* (Cf), *Camelus bactrianus* (Cb), and *Vicugna pacos* (Vp). The CdPMAP-23 was highly similar to CfPMAP-23 isoforms X1 and X2 (95.24% identity) and VpPMAP-23 (72.73% identity) (**Supplementary Figure 1A**). CdCATH showed the greatest similarity to Cb CATH-6, Cf CATH-6, Cd CATH-6 (100% identity), and VpCATH-6 (89.66%) (**Supplementary Figure 1B**). Additionally, CdPG-3 exhibited a high degree of identity with CdPMAP-37 (91.43%) and was more than 85% similar to other alignments (**Supplementary Figure 1C**).

**Table 1:** Prediction of cathelicidins AMPs from dromedary camels using BlastP in the NCBI database, Signal P-5.0, peptide cutter-Expasy tool, and The Antimicrobial Sequence Scanning System (AMPA) database to detect antimicrobial active regions with a threshold value of 0.225.

Accession NO.	Name	Predicted cathelicidin peptide sequence	Signal P	Peptide cutter site	AMPA
NP_004336.4	LL-37 (reference peptide)	LLGDFFRKSKEKIGKEFKRIVQRIKDFLRNLVPRTES	0.45	1st valine in Exon4	SKEKIGKEFKRIVQRI
XP_031326097.1	CdPMAP-23-like	KIINLPWRPPPRKRPIRVIYV	0.9678	1^st^ valine in Exon4	RPPPRKRPIRVIYV
KAB1264031.1	CdProtegrin-3	GLFGRIRDSIRNRVNRVRDKVGKVIGYIGDKIRPG	none	1^st^ valine in Exon4	SIRNRVNRVRDKVGKVIGY
XP_031326999.1	Cdcathelin-like	GFFKKARNKLKNAWRKVGPIVGPLLTFFG	0.0018	1^st^ valine in Exon4	FKKARNKLKNAWRKVGPIVG

#### 3.1.2 Physicochemical properties of identified peptides

The physicochemical properties of LL-37, CdPMAP-23, CdPG-3, and CdCATH were estimated using APD3 and the Compute pI/MW tool—Expasy (**Table 2**). The net charge was +6 for CdPMAP-23 and +7 for the remaining peptides. CdCATH with a molecular weight of 3290.98 Dalton had the highest hydrophobic ratio (45%), which may contribute to high antibacterial activity. The shortest peptide length was CdPMAP-23 (21 residues), with a molecular weight of 2612.251 kDa and a hydrophobic ratio of 38%. CdPG-3 showed a length of 35 residues, with a 31% hydrophobic ratio and a molecular weight of 3981.67 kDa. The isoelectric point varied from 10.61 (LL-37), 11.63 (CdPG-3), 12.01 (CdPMAP-23), to 12.04 (CdCATH).

**Table 2:** Prediction of physicochemical properties including from left to right: peptide length, net charge, hydrophobic ratio, molecular weight, and isoelectric point using the Antimicrobial Peptide Database Calculator and Predictor (APD3) and the Compute pI/MW tool—Expasy.

Peptide	Peptide length	Net charge	Hydrophobic ratio	Molecular weight	Isoelectric point
LL-37 (reference peptide)	37	6	35%	4493.32	10.61
CdPMAP-23	21	6	38%	2612.251	12.01
CdPG-3	35	7	31%	3981.668	11.63
CdCATH	29	7	45%	3290.981	12.04

**Figure 2:**
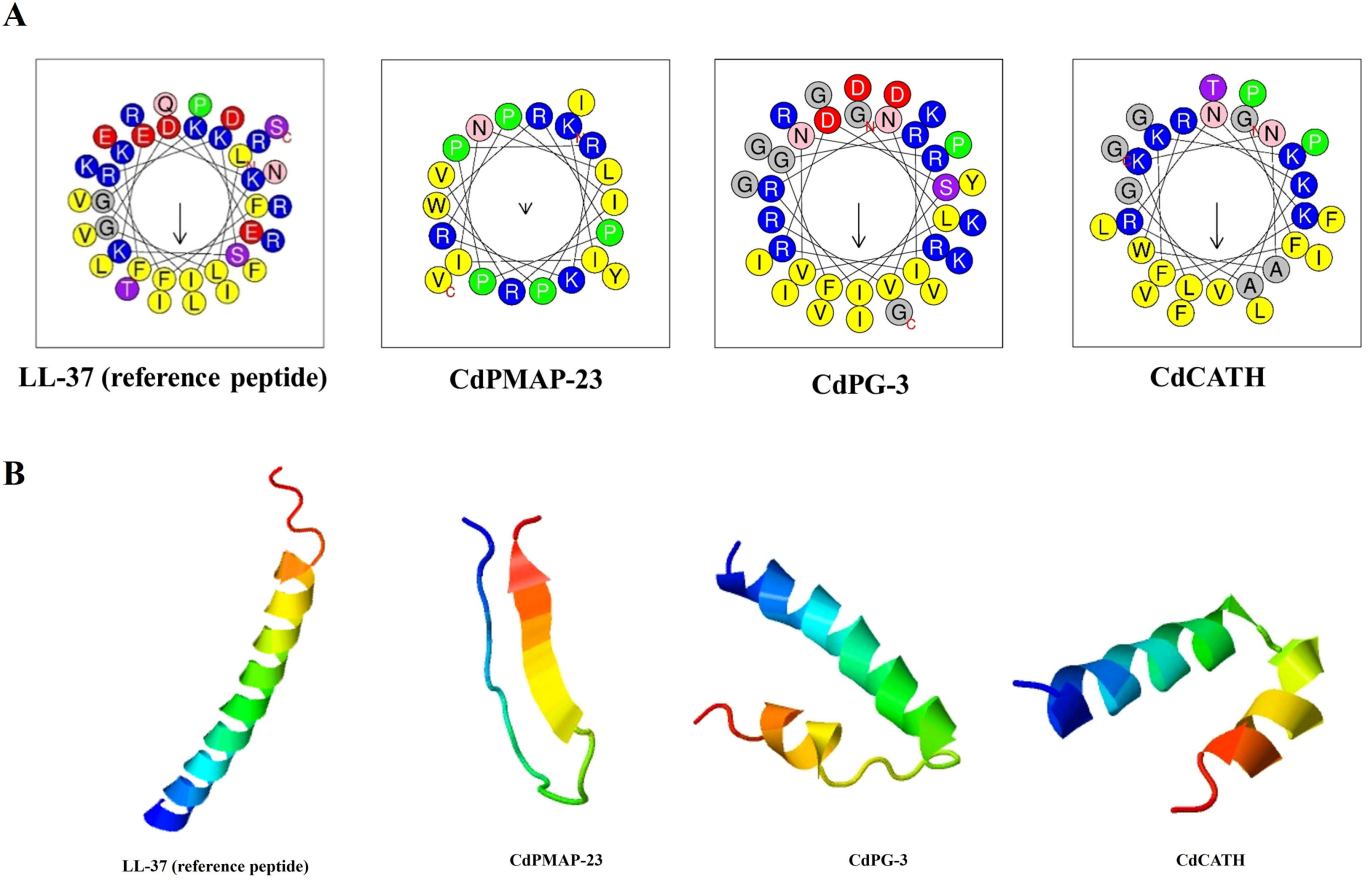
Structural characterization of identified peptides. **(A)** Helical wheel projection of α-helical peptides using HeliQuest analysis. Residues highlighted in yellow are hydrophobic, while those shown in blue are cationic. Polar acidic residues, specifically aspartic acid (D) and glutamic acid (E), are represented in red. Residues containing amine groups, asparagine (N) and glutamine (Q), are presented in pink, whereas hydroxy-amino acids, serine (S) and threonine (T), are illustrated in purple. Glycine residues are displayed in grey. The arrow showed the direction of the hydrophobic moment<μH>. **(B)** Prediction of the 3D structure of peptides using I-TASSER.

The HeliQuest database was utilized to identify the α-helical 2D structures of the identified peptides. The CdCATH and CdPG-3 demonstrated a more pronounced amphipathic character compared to CdPMAP-23, based on the amphipathic moment<µH> values 0.509, 0.493, and 0.114, respectively (**Figure 2A**). This prediction suggested that the CdCATH and CdPG-3 peptide sequences may adopt an α-helical conformation. Therefore, the tertiary structure was predicted using the I-TASSER server. CdPG-3 and CdCATH comprised a double α-helical structure linked by a central hinge. In contrast, CdPMAP-23 consisted of a random coil structure, as predicted by this tool (**Figure 2B**).

#### 3.1.3 Evaluation of the membrane-binding capability of identified peptide

The Boman index estimates the potential of protein binding. A high index value (>2.48) suggests a strong binding capability, while a low index (≤1) shows fewer side effects (33). The result showed that CdPG-3 presented the highest index (3.13), followed by LL-37 (2.99), CdPMAP-23 (2.02), whereas CdCATH has the lowest index (0.82). According to these unexpected values, AMPs often cannot interact with proteins directly but instead disrupt and penetrate the plasma membrane. The TMHMM server was used to evaluate the probability that the peptides would translocate across lipid membranes based on their cellular localization. The results showed that CdPMAP-23 and CdPG-3 were located outside cell membranes, except CdCATH, which was located inside the cell (**Supplementary Table 7**).

#### 3.1.4 Prediction of biological properties of identified peptides

The identified peptides and their probability scores (SVM, ANN, and RF of natural and synthetic) are listed in **Table 3**. The antimicrobial properties of natural antimicrobial peptides (AMPs) were predicted to be high for the three identified peptides, with probability scores exceeding 0.85 across all models. All identified peptides demonstrated significant antiviral and antifungal activity with low SVM scores. All peptides were predicted to be non-hemolytic (**Supplementary Table 8**). The composition of residues associated with hemolysis is presented in **Supplementary Table 9**. CdPMAP-23 and CdPG-3 exhibited modest hydrophobicity (33–34%) and a higher arginine content, whereas CdCATH had the highest hydrophobicity (55.17%), with a lower arginine level and a moderate amount of tryptophan. These differences suggest variations in the potential hemolytic activity among the peptides.

**Table 3:** Prediction of antimicrobial activity using three machine-learning algorithms in the CAMP_R4_ database for natural and synthetic peptides: Support Vector Machine (SVM), Artificial Neural Network (ANN), and Random Forest (RF).

Peptide	Natural AMP			Synthetic AMP		
	SVM	ANN	RF	SVM	ANN	RF
LL-37 (reference peptide)	0.64	0.81	0.66	0.32	0.38	0.40
CdPMAP-23	0.94	0.93	0.85	0.99	0.98	0.98
CdPG-3	0.90	0.96	0.98	0.92	0.69	0.82
CdCATH	0.99	0.98	0.99	0.99	0.96	0.97

They give a probability score (0 to 1) for the prediction; the higher the probability, the greater the possibility of the peptide being antimicrobial.

### 3.2 Experimental analysis

#### 3.2.1 Circular dichroism spectroscopy

**Figure 3:**
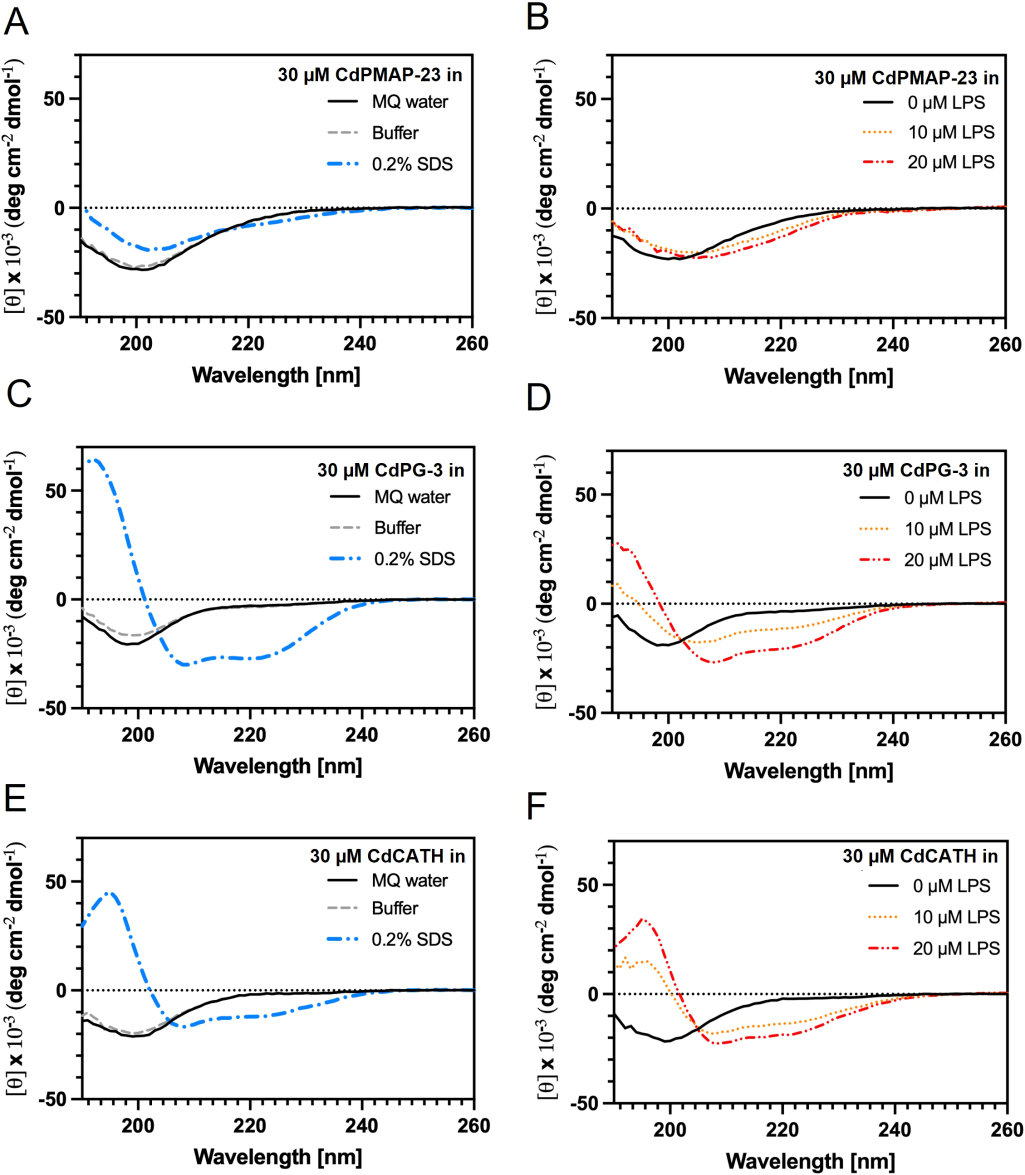
Analysis of conformational changes of identified peptides in LPS and SDS by circular dichroism. CD spectra of CdPMAP-23 **(A, B)**, CdPG-3 **(C, D)**, and CdCATH **(E, F)** peptides were applied with a 190–260 nm of spectral range, under various conditions. Conformational changes were followed in the presence of MQ water **(A, C, E)**, buffer conditions **(A, C, E)**, 0.2% SDS **(A, C, E)**, and 10 or 20 µM LPS **(B, D, F)**. The spectra were recorded using a 10 mM sodium phosphate solution adjusted to pH 7.4 at RT under quiescent conditions.

To study the structural properties of the three identified peptides, circular dichroism (CD) spectroscopy experiments were applied with a 190–260 nm of spectral range (**Figure 3**). At a 30 µM concentration, all peptides exhibited random coil-like spectra and unstructured properties in both Milli-Q water and phosphate buffer at pH 7.4 conditions (**Figures 3A, C, E**). After adding 0.2% SDS, the CdPG-3 and CdCATH peptides showed a-helical content up to 77.6 ± 0.0% and 39.8 ± 0.0% for the respective peptides (**Table 4**). The presence of 0.2% SDS to CdPMAP-23 only induced minor effects (**Figure 3A**). Two titration steps were performed with LPS to explore the effects of LPS on the secondary structures (**Figures 3B, D, F**). Similar to the spectra after the addition of SDS, 20 µM LPS also caused conformational changes of CdPG-3 and CdCATH peptides into a-helical conformations to the extent of 60.5 ± 0.2% and 56.2 ± 0.4%, respectively (**Table 4**).

**Table 4:** α-helical content at different conditions determined for CdPMAP-23, CdPG-3, and CdCATH peptides.

Experimental conditions	30 μM CdPMAP-23 (% α-helical content)	30 μM CdPG-3 (% α-helical content)	30 μM CdCATH (% α-helical content)
MQ water	22.8 ± 0.1	17.1 ± 0.1	14.2 ± 0.6
Buffer pH 7.4	23.0 ± 0.1	18.5 ± 0.2	14.2 ± 0.3
0.2% SDS	29.0 ± 0.5	77.6 ± 0.0	39.8 ± 0.0
10 μM LPS	31.5 ± 0.7	37.9 ± 0.7	43.4 ± 3.3
20 μM LPS	37.7 ± 0.1	60.5 ± 0.2	56.2 ± 0.4

#### 3.2.2 Antimicrobial activity assessed by CFU assays

Next, the antibacterial effects of LL-37 (reference peptide), CdPMAP-23, CdPG-3, and CdCATH against multiple bacterial strains, including *Staphylococcus aureus* (*S. aureus)*ATCC 25923, *Methicillin-resistant Staphylococcus aureus* (*MRSA*) ATCC 700699, *Escherichia coli* (*E. coli*) ATCC 25922, *Escherichia coli* multidrug resistant (*E. coli* MDR), and *Klebsiella pneumoniae* (*K. pneumoniae*) ATCC 1705 and ATCC 1706, across a concentration range of 1.25 to 160 μM were evaluated.

**Figure 4:**
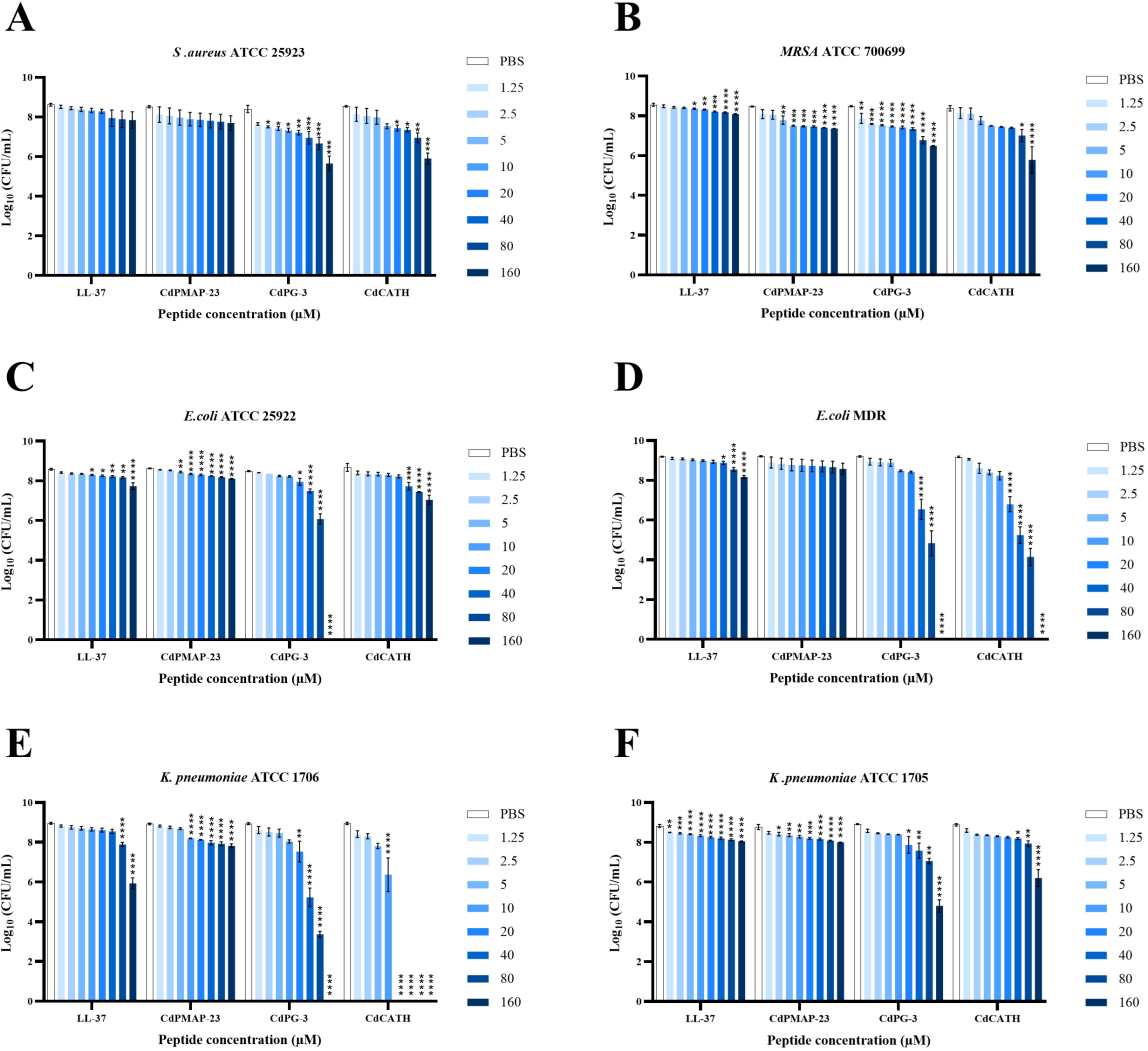
Antibacterial activity of identified peptides against Gram-positive and Gram-negative bacteria. Colony forming units (CFU) of **(A)***S. aureus* ATCC 25923, **(B)***MRSA* ATCC 700699, **(C)***E. coli* ATCC 25922, **(D)***E. coli* MDR, and **(E, F)***K*. *pneumoniae* ATCC 1706 and ATCC 1705 were presented to eight concentrations of LL-37, CdPMAP-23, CdPG-3, and CdCATH for 3 h at 37°C. After serial dilutions and overnight incubation on LB agar, the colonies of surviving bacteria were counted, (PBS as negative control), data is presented as Mean Log_10_ CFU/ml, P value: 0.05>(*), 0.01>(**), 0.001> (***), 0.0001>(****).

For *S. aureus* ATCC 2592, LL-37 and CdPMAP-23 did not significantly reduce bacterial growth at any concentration. However, CdPG-3 showed a notable effect: a reduction of 0.9 to 1 log_10_ for 2.5 µM to 10 µM (p<0.05), 1.1 log_10_ for 20 µM (p<0.01), 1.2 log_10_ for 40 µM, and 1.3 log_10_ for 80 µM (p<0.001). The highest concentration of 160 µM led to a 2 log_10_ decrease (p<0.0001). For CdCATH, concentrations of 20 µM and 40 µM resulted in a 1 to 1.2 log_10_ reduction (p<0.05), while 80 µM caused a 1.5 log_10_ decrease (p<0.01). At 160 µM, growth decreased by 2.5 log_10_ (p<0.0001) (**Figure 4A**).

Regarding *MRSA* ATCC 700699, LL-37 had a notable effect starting at 10 µM, reducing growth by 0.2 log_10_ (p<0.05) and reaching 0.48 log_10_ at 160 µM (p<0.0001). CdCATH showed a different response, with significant reductions beginning at 5 µM (0.56 log_10_, p<0.01) and a 1.1 log_10_ reduction at 160 µM (p<0.0001). CdPG-3 was more effective at lower doses, showing a 0.4 log_10_ decline at 1.25 µM (p<0.01) and up to 1.6 log_10_ at 80 and 160 µM (p<0.0001). CdCATH, however, had no significant effects at lower concentrations until 80 µM, where it caused a 1.2 log_10_ reduction (p<0.05), increasing to 2.1 log_10_ at 160 µM (p<0.0001) (**Figure 4B**).

For *E. coli* ATCC 25922, CdPG-3 was most effective at inhibiting bacterial growth, showing a 0.5 log_10_ decrease at 20 µM (p<0.05), 1 log_10_ at 40 µM (p = 0.0001), and 2.3 log_10_ at 80 µM (p<0.0001). Complete inhibition occurred at 160 µM. CdCATH started showing significant effects at 40 µM with a 1 log_10_ reduction (p<0.001), increasing to 1.3 log_10_ at 80 µM and 1.6 log_10_ at 160 µM (p<0.0001). CdPMAP-23 showed growth reduction beginning at 5 µM, reaching 0.5 log_10_ at 160 µM (p<0.0001) (**Figure 4C**).

Interestingly, CdCATH and CdPG-3 showed significant growth inhibition against *E. coli* MDR at higher concentrations starting from 20 µM and 40 µM (2.1 log_10_, p<0.0001), respectively. In contrast, CdPMAP-23 has no effect against *E. coli* MDR. For LL-37, significant inhibition of bacterial growth began at 40 µM (0.27 log_10_ reduction, p< 0.05) and increased at 80 µM (0.61 log_10_ reduction, p<0.0001) and 160 µM (0.99 log_10_ reduction, p<0.0001) (**Figure 4D**).

*K. pneumoniae* ATCC 1706 showed a significant reduction in growth with LL-37, decreasing by 1.04 log_10_ at 80 µM (p<0.0001) and 2.85 log_10_ at 160 µM (p<0.0001). CdPMAP-23 had no notable effect until 10 µM, achieving a maximum reduction of 1.08 log_10_ at 160 µM. CdPG-3 resulted in a 1 log_10_ reduction at 20 µM (p<0.05), maintaining a decrease of 5 log_10_ at 80 µM (p<0.0001). CdCATH showed a 1.4 log_10_ reduction at 10 µM (p<0.01) and a significant 5.5 log_10_ decrease at 20 µM (p<0.001). At concentrations of 40 µM and above, bacterial growth was completely suppressed (**Figure 4E**).

*K. pneumoniae* ATCC 1705 is a strain recognized for producing the carbapenemase enzyme KPC, which makes it resistant to carbapenem antibiotics. Significant inhibition of *K. pneumoniae* growth was observed at all tested concentrations of LL-37, with higher concentrations leading to greater inhibition. CdPMAP-23 demonstrated significant activity starting at 2.5 µM, resulting in a minor reduction in bacterial growth up to 160 µM, which caused a 0.8 log_10_ reduction. This strain also showed a notable reduction in growth when exposed to CdPG-3 and CdCATH at 20 µM, achieving a 3.5 log_10_ reduction at 160 µM for CdPG-3 (p<0.0001) (**Figure 4F**).

#### 3.2.3 Sytox green uptake assay

**Figure 5:**
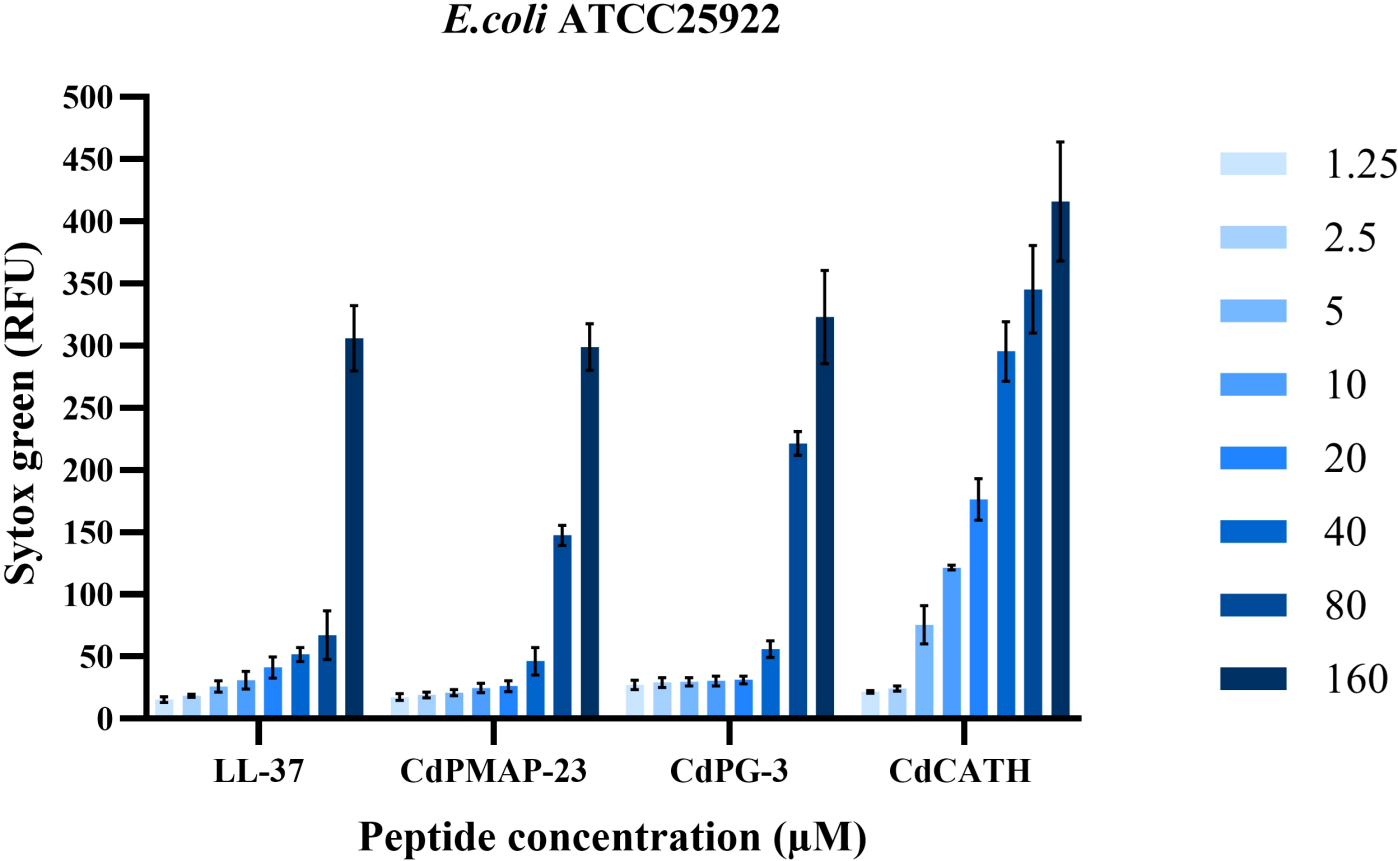
Membrane permeabilization properties of identified peptides on *E. coli* ATCC 25922. *E. coli* (5 × 10^7^ CFU/mL) was incubated for 30 min at 37 °C with LL-37, CdPMAP-23, CdPG 3, and CdCATH at different concentrations. After centrifugation and washing, the bacteria were stained with 3 μM SYTOX Green for 5 minutes, and fluorescence was measured at an excitation wavelength (λ_ex_) of 504 nm and an emission wavelength (λ_em_) of 523 nm using a Varioskan LUX reader. Results are presented as the mean ± SEM (N = 3).

The capacity of the peptides LL-37 (reference peptide), CdPMAP-23, CdPG-3, and CdCATH to induce membrane leakage in *E. coli* ATCC 25922 was estimated using Sytox Green, a permeability marker that detects nucleic acids at various peptide concentrations. The CdCATH exhibited the highest activity, with fluorescence intensities increasing significantly from 5 µM to 160 µM, indicating strong membrane disruption in *E. coli*. For CdPG-3, the highest leakage on the *E. coli* membrane was observed at concentrations ranging from 40 µM to 160 µM, while a minimal permeability was observed within the concentration range from 1.25 to 20 µM. Similarly, CdPMAP-23 exhibited high membrane permeability between 80 and 160 µM concentrations. However, LL-37 induced membrane permeability in the range of 1.25–160 µM (**Figure 5**).

#### 3.2.4 Transmission and scanning electron microscopy

**Figure 6:**
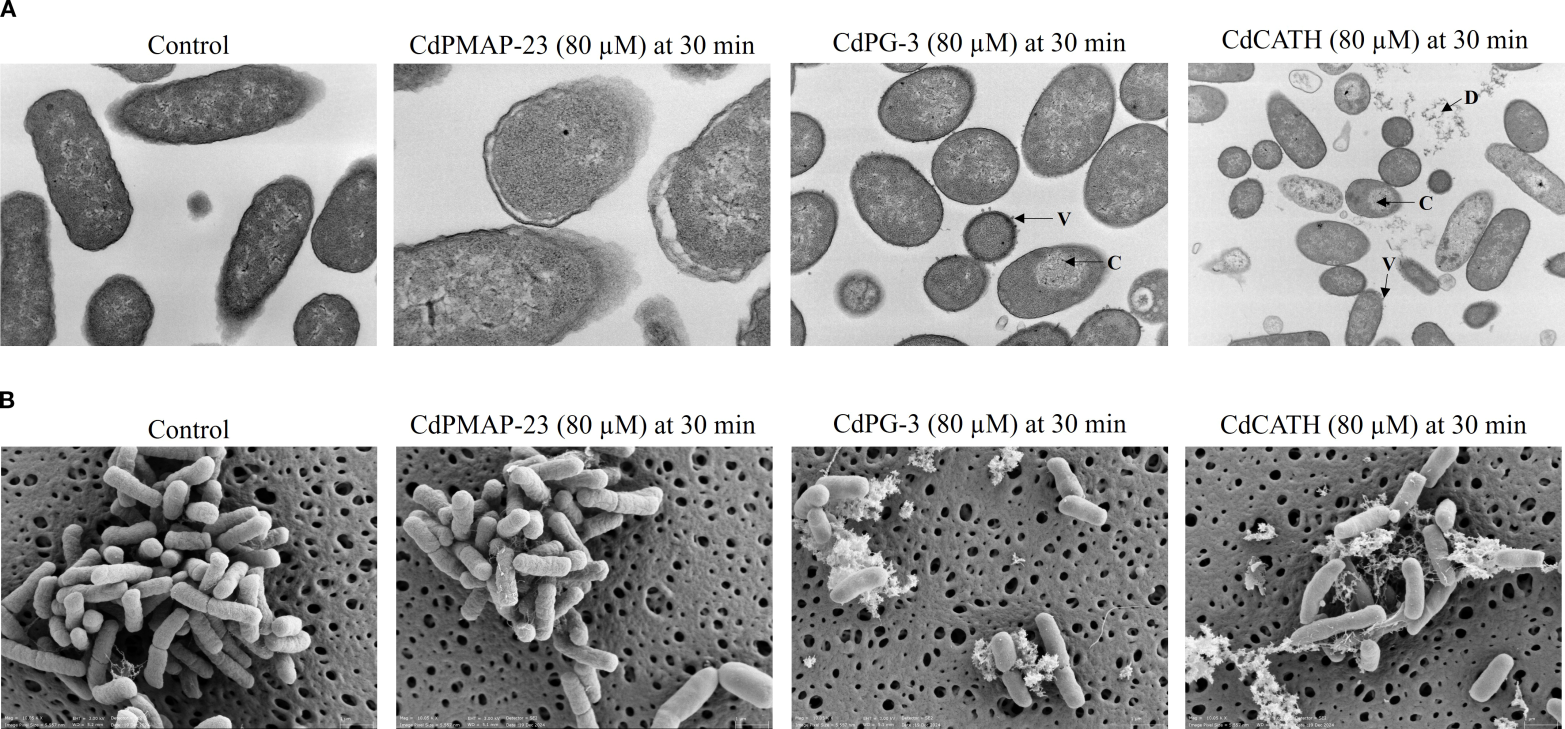
Morphological changes induced by the identified peptides on *E. coli* ATCC 25922. CdPMAP-23, CdPG-3, and CdCATH caused morphological changes to *E*. *coli* ATCC 25922. *E*. *coli* (5 × 10^8^ CFU/mL) was cultured, treated with 80 µM of each peptide at 37 °C for 30 minutes, and prepared for both microscopy techniques. **(A)** Representative images illustrate the intracellular alterations observed by TEM after samples were fixed, stained, and imaged with a Tecnai Spirit G2 Bio TWIN Electron microscope. **(B)** Visualization of surface morphological changes by SEM after samples were fixed, coated with platinum, and imaged using an Ultra 55 field-emission scanning electron microscope. V, vesicles; D, DNA leakage; C, clustered DNA.

Transmission Electron Microscopy (TEM) was utilized to investigate the intracellular changes caused by CdPMAP-23, CdPG-3, and CdCATH peptides in *E. coli* ATCC 25922. The untreated bacteria appeared intact, featuring well-preserved membranes and cytoplasmic contents. There were no indications of damage or disruption in the cellular structure, and the distribution of DNA and ribosomes within the cytoplasm was consistent. After treatment with CdPMAP-23, the cells showed significant membrane disruption. The bacterial cells appeared swollen, with clear leakage of their internal contents, and some cells looked like they had undergone lysis. In contrast, treatment with CdPG-3 resulted in partial lysis, swelling, membrane damage, and variations in the cytoplasm. Furthermore, some inclusion bodies, such as vesicles in certain cells, likely arose from internal disruptions or localized injuries. The cells experienced extensive damage from CdCATH peptides, with most bacterial cells showing severely compromised structures, including broken membranes, DNA damage, and a significant loss of intracellular components (**Figure 6A**).

Scanning Electron Microscopy (SEM) was used to image possible alterations in the surface bacterial membrane. The control showed intact, healthy bacterial cells. The cells had a smooth surface morphology, with no noticeable surface defects or irregularities. Treatment with CdPMAP-23 resulted in significant surface damage to the bacterial cells. Certain cells seemed to be wrinkled, irregularly shaped, and swollen. CdPG-3 induced irregularities on bacterial cell surfaces and lead to pore formation. It appeared to be more swollen compared to CdPMAP-23. CdCATH treatment resulted in the most significant damage to bacterial cells. Many cells were completely disrupted, leading to the leakage of cellular components. The surfaces of the cells showed signs of lysis, with materials scattered and pores formed (**Figure 6B**). Overall, it appears that CdCATH peptides caused the most damage to bacterial membranes and induce cell lysis, followed by CdPG-3 and CdPMAP-23 with different mechanisms of action.

#### 3.2.5 Hemolysis assay

The hemolytic effects of the novel camel AMPs were studied at varying concentrations in red blood cells from humans, camels, goats, and chickens.

**Figure 7:**
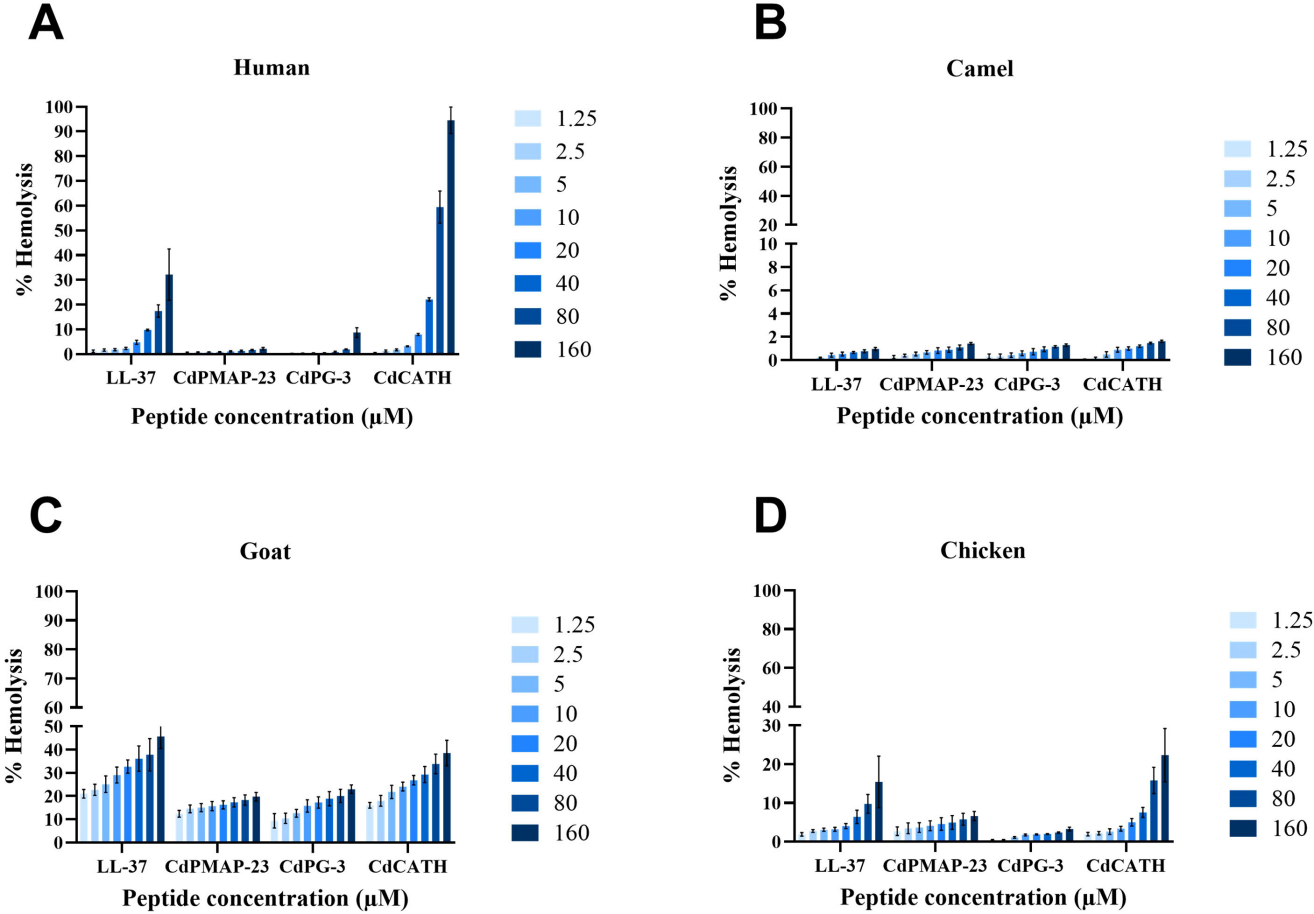
Hemolytic activity of identified peptides against erythrocytes from different species. Hemolysis assay of **(A)** Human RBCs, **(B)** Dromedary camel RBCs, **(C)** Goat RBCs, and **(D)** Chicken RBCs. RBCs were treated for 1 hour with LL-37, CdPMAP-23, CdPG-3, and CdCATH at different concentrations. The hemolytic activity was estimated by determining the hemoglobin release absorbance at 540 nm; ≤ 2% is non-hemolytic, between 2–5% is slight hemolysis, between 5–10% is moderate hemolysis, and > 10% is severe hemolysis. Results are presented as the mean ± SEM (N = 3).

In human blood, CdPMAP-23 presented almost no hemolysis for all concentrations tested, showing values below 5%, indicating minimal interaction with erythrocytes. Similarly, CdPG-3 also presented no hemolysis, showing moderate hemolysis in a higher concentration of 160 µM (8.7%) but never above 10%. By contrast, LL-37 showed a level of hemolysis that increased gradually in percentage as the concentration increased. There was slight hemolysis in concentrations from 1.25 µM (1.2%) to 20 µM (4.8%) and moderate hemolysis in concentrations 40 µM (9.8%) but severe hemolysis at concentrations 80 µM (17.5%) and 160 µM (32.2%). However, the human blood showed the highest sensitivity against CdCATH, which presented a substantial, dose-related increase in percentage hemolysis, clearly showing more than 50% hemoglobin release at the 80 µM and 160 µM concentrations tested. However, there was slight hemolysis at a concentration from 1.25 µM (0.6%) to 10 µM (3.2%) and moderate hemolysis at 20 µM (8%) (**Figure 7A**). In camel blood, as shown in **Figure 7B**, all peptides tested were non-hemolytic > 2% in concentration from 1.25 µM to 160 µM. For goat blood, the results indicated that the percentage of hemolysis increased with the increase in concentration of each peptide. Almost all peptides tested in this study showed severe hemolysis > 10% in concentration from 1.25 µM to 160 µM (**Figure 7C**). In chicken blood, CdPMAP-23 demonstrated moderate hemolysis at various concentrations. In contrast, CdPG-3 showed minimal hemolytic activity, staying under 5% even at the highest concentration tested. As the concentration increased, LL-37 and CdCATH significantly increased hemolytic activity, reaching approximately 20–30% hemolysis at the highest concentration of 160 μM (**Figure 7D**).

## 4 Discussion

In this study, three novel cathelicidin peptides were identified *in-silico*: CdPMAP-23, CdPG-3, and CdCATH from the dromedary camel. These peptides were predicted based on species homology and specific characteristics, such as net charge and hydrophobicity, which suggest their strong potential for membrane interaction and disruption. They share important features of the cathelicidin peptide family. This includes having the same highly conserved pro-region at the N-terminal cathelin-like domain (CLD) with four cysteines.

Structural analysis showed that CdPG-3 and CdCATH have an amphipathic α-helical structure, consisting of two α-helices linked by a central hinge. This structure is frequently observed in natural AMPs, such as chicken CATH-1, -2, and -3, and Porcine PMAP-36 and PMAP-23 (39–43). However, CdPMAP-23 is a random coil, while CdPG-3 and CdCATH can adopt α-helical conformations in membrane-mimetic conditions. This structural difference may explain why CdPMAP-23 reduced antibacterial activity, as its less hydrophobic conformation likely impairs its interaction with microbial lipid membranes. A previous study has shown that AMPs exhibit structural flexibility, existing as unstructured states and folding into α-helical or β-sheet conformations under membrane-mimetic conditions (44). This structural flexibility is crucial to antimicrobial activity, as helical structures enable membrane insertion and disruption, thereby enhancing their bactericidal activity. We found that the behavior of CdPG-3 and CdCATH was quite similar to that of other cathelicidin-derived peptides; they would assume an amphipathic α-helical structure and thus display a strong affinity for anionic lipid bilayers upon interaction (45). CdPG-3 and CdCATH peptides showed an increase in α-helical content when exposed to LPS. These results are consistent with a study that suggests that LPS binding improves the secondary structures of AMPs, thereby enhancing their interaction with bacterial membranes (46).

In this study, *in-silico* prediction of antimicrobial activity revealed that CdCATH and CdPG-3 have the highest antimicrobial potential compared to CdPMAP-23. This finding is consistent with the experimental results, where CdCATH and CdPG-3 demonstrated the highest antimicrobial activity against all tested bacteria, compared to CdPMAP-23. Interestingly, CdCATH and CdPG-3 showed significant growth inhibition against multidrug-resistant strains, including *E. coli* MDR and *MRSA* ATCC 700699. This finding aligns with studies on protegrin-1 (PG-1) analogs, which demonstrated potent activity against Gram-positive pathogens like *S. aureus* ATCC 29213 and penicillin-resistant *S. aureus* 209P, with lower MIC values (1 µM, 0.5 µM), and Gram-negative bacteria, such as *E. coli* ATCC 25922, with MICs ranging from 0.12 - 2 µg/ml (0.05–0.84 µM) (47, 48). Moreover, VicBac cathelicidin peptide from Alpaca *Vicugna pacos* had antibacterial activity against reference and *E. coli* MDR strains with MICs ranging from 0.25 – 8 µM (31). A previous study indicated that horse CATH-1 was effective against Gram-negative bacteria (MICs 0.96–3.82 µM) and Gram-positive bacteria (MICs1.91-7.64 µM), with higher MICs of 15.91 µM observed only for the *MRSA* strain (49).

These differences in effectiveness may be attributed to the structural variations among different cathelicidin peptides, as well as the specific bacterial strains and experimental conditions employed. The differences in susceptibility levels among the various bacterial strains may correlate with variations in membrane composition or permeability, which are presumed to result in altered peptide binding and activity. These conclusions reinforce the potential of CdCATH and CdPG-3 peptides for antibacterial therapy in relation to the global emerging challenge of antibiotic resistance. The fact that these two peptides were particularly effective against antibiotic-resistant strains, such as *MRSA* ATCC 700699 and *E. coli* MDR, further supports their use as a potential therapeutic agent in treating these infections.

However, our research revealed that CdPMAP-23 was ineffective against *S. aureus* ATCC 25923. This finding disagrees with a study showing that porcine PMAP-23 exhibited strong antibacterial activity against *S. aureus* ATCC 25923, *S. aureus* ATCC 29213, and *S. epidermidis* ATCC 12228, with MICs of 2 µM, 4 µM, and 2 µM, respectively (50). The effectiveness of porcine PMAP-23 against *S. aureus* is attributed to its higher content of hydrophobic amino acids like leucine, valine, and phenylalanine, which enhance membrane penetration. In contrast, CdPMAP-23 has these replaced by more polar amino acids, increasing its positive charge but reducing hydrophobicity. Differences in assay sensitivity and bacterial incubation duration can also affect results. Additionally, physiological conditions, such as culture media, can impact antibacterial activity, as demonstrated by Porcine PMAP-36, which showed varying effectiveness against *E. coli* ATCC 25922 in different media (51). CdPMAP-23 showed a dose-dependent inhibitory effect on *E. coli* ATCC 25922, aligning with various studies that indicate differing MIC for porcine PMAP-23 against different *E. coli* strains: 2.5 µM for *E. coli* BW25113, 32 µM and 43.22 µM for *E. coli* ATCC 25922 (52–54).

In this study, the three peptides gradually enhanced the permeability of the inner membrane, causing damage to the internal structures and surface morphology of *E. coli* ATCC 25922. Notably, CdCATH and CdPG-3 had the greatest effect compared to CdPMAP-23. These results were in accordance with those obtained from the CFU assay in this study, which employed different mechanisms. The variations in membrane permeabilization by CdCATH, CdPG-3, and CdPMAP-23 in the present study can be attributed to differences in their amino acid sequences and hydrophobic and amphipathic properties, which determine their interactions with bacterial lipid membranes. This finding is consistent with studies that indicated the hydrophobic moment and cationic amphiphilic AMPs are electrostatically attracted to a negatively charged bacterial surface layer and incorporated into the hydrophobic part of the membrane, causing damage and disintegration (55, 56).

Hemolysis measures how cationic peptides affect mammalian cell membranes, indicating the safety of antibiotics (57). The *in-silico* prediction of hemolytic activity suggested that all identified peptides were likely non-hemolytic based on statistical analysis, suggesting they are safe despite slight variations. Computational tools mainly depend on statistical and physicochemical models and can provide valuable preliminary predictions. However, these models often do not align well with experimental data on antimicrobial or hemolytic activity, particularly for short, highly cationic peptides. Therefore, it is essential to conduct experimental validations (58). However, experimentally, all peptides tested were non-hemolytic to camel RBCs. Research indicates that camel erythrocytes are resistant to osmotic lysis due to their unique membrane structure, which contains high levels of cholesterol and specific phospholipids. As a result, camel RBCs are more resistant to hemolysis in hypotonic environments compared to those of humans, sheep, and goats. Also, they can swell to twice their normal volume before rupturing, whereas the RBCs of other species only expand to 150% of their standard size (59). Notably, CdCATH was the most prevalent cause of RBC lysis in humans, goats, and chickens, exhibiting a dose-dependent effect. The primary criteria for estimating hemolytic activity are net charge and hydrophobicity (60, 61). Commonly, the hemolytic activity increases when the hydrophobicity of amphipathic α-helical peptides is increased (62). In the current study, hydrophobicity was linked with hemolytic activity, which is in line with other findings, since the hemolytic activity against erythrocytes increased with the hydrophobicity of peptides (63, 64). Previous studies have shown that higher hydrophobicity peptides enter more deeply into the hydrophobic core of the RBC membrane, creating pores or channels that cause stronger hemolysis (62, 65). The second criterion considered for estimating hemolytic activity is the amino acid composition commonly associated with hemolysis (e.g., Arginine, Lysine, Tryptophan) and the proportion of cationic and hydrophobic residues (66). CdPMAP-23 and CdPG-3 were predicted to have a high arginine percentage (19-20%), which contributes to increasing the electrostatic attraction towards negatively charged membranes. In contrast, the CdCATH has a higher percentage of lysine (17.24%), which is attributed to the peptide’s high cationic charge character and its high affinity towards membranes. Stimulating deeper membrane insertion, CdPMAP-23 demonstrated a relatively higher tryptophan content, which may affect its ability to disrupt membranes. As a result, it can effectively interact with the membranes of microbes while limiting damage to host cells. Of interest are also the findings that the CdCATH peptide exhibited the highest hydrophobicity, which likely explains its superior activity compared to CdPG-3 and CdPMAP-23 in the study.

Despite the successes of this research, several limitations remain: first, this study focused on the antibacterial activity against six bacterial strains; however, future studies should include a broader range of bacteria and fungi to draw more comprehensive conclusions. Second, in addition to their antimicrobial properties, cathelicidins also exhibit diverse immunomodulatory activities; future studies should include assays for these activities. Third, the expression of these peptides across different camel tissues should be carefully mapped. Fourth, peptide sequences can be chemically modified to enhance stability and reduce hemolysis. finally, our investigation was limited to identifying cathelicidin antimicrobial peptides. Future research could also aim to identify and characterize other types of AMPs, such as defensins found in dromedary camels.

## 5 Conclusion

Our study successfully identified three novel cathelicidin peptides from dromedary camels: CdPMAP-23, CdPG-3, and CdCATH, using both experimental and *in-silico* approaches. Both CdCATH and CdPG-3 showed significant antibacterial effects against all evaluated Gram-positive and Gram-negative strains. CdCATH and CdPG-3 resulted in the highest membrane leakage and considerable damage to the internal structures and surface morphology of *E. coli* ATCC25922, followed by CdPMAP-23. These findings enhance our understanding of the unique features of dromedary camels, their innate immune system, and its components. Also, highlight the potential of CdCATH and CdPG-3 as antimicrobial peptides against antibiotic-resistant strains such as *MRSA* ATCC 700699 and *E. coli* MDR, paving the way for further research in this area.

## Data Availability

The original contributions presented in the study are included in the article/**Supplementary Material**. Further inquiries can be directed to the corresponding author.
