# Supplementary material for: Identification and characterization of novel antimicrobial peptides from Camelus dromedarius: a combined bioinformatics and experimental study
Source: Front Immunol. 2026 Jan 22;17:1745714. doi: 10.3389/fimmu.2026.1745714 (PMC12872550; doi:10.3389/fimmu.2026.1745714)
Supplement: Supplementary file 1 [file DataSheet1.docx]

**Supplementary Materials**

1. **Extraction and Enrichment of Leukocyte-Derived Polypeptides**

Blood samples were processed quickly to lyse RBCs by diluting them with distilled water and PBS, following the previous study (1). The leukocyte pellet was suspended in a 60% acetonitrile solution with trifluoroacetic acid (TFA) and incubated overnight, then freeze-dried. The lyophilized extract was dissolved in TFA-water, centrifuged, and the protein concentration was determined. Polypeptides were enriched on Sep-Pak C18 columns, eluted with acetonitrile, and lyophilized for storage. For LC-MS/MS Analysis, polypeptides were dissolved, reduced, alkylated, and digested with trypsin. After cleaning, peptides were separated on a C18 column, and mass spectra were obtained using a Q Exactive HF instrument for further analysis.

**Supplementary Table 1:** Antibiotic Susceptibility of *Staphylococcus aureus* ATCC 25923

| **Antibiotic** | **MIC (µg/ml)** | **Result** | **Break P (S)** | **Break P (S)** |
| --- | --- | --- | --- | --- |
| Penicillin | ≤0.12 | S | ≤ 0.125 | > 0.125 |
| Minocycline |  | S |  |  |
| Tigecycline |  | S |  |  |
| Midecamycin |  | S |  |  |
| Meleumycin |  | S |  |  |
| Clarithromycin |  | S |  |  |
| Acetylspiramycin |  | S |  |  |
| Rokitamycin |  | S |  |  |
| Teicoplanin | ≤2 | S | ≤2 | >2 |
| Dalbavancin |  | S |  |  |
| Trimethoprim- sulfamethoxazole | ≤2/38 | S | ≤2/38 | >4/76 |
| Lincomycin |  | S |  |  |
| Daptomycin | ≤0.5 | S | ≤1 | >1 |
| Tedizolid |  | S |  |  |
| Erythromycin + Lincomycin | ≤4/0.5 | * |  |  |
| Cefoxitin | ≤4 | * |  |  |
| Rifampicin | ≤0.5 | * | ≤0.06 | >0.06 |
| Tetracycline | ≤1 | S | ≤1 | >2 |
| Doxycycline | ≤4 | S | ≤1 | >2 |
| Erythromycin | ≤0.5 | S | ≤1 | >2 |
| Azithromycin | ≤1 | S | ≤2 | >2 |
| Roxithromycin |  | S |  |  |
| Josamycin |  | S |  |  |
| Dirithromycin |  | S |  |  |
| Vancomycin | ≤1 | S | ≤2 | >2 |
| Oritavancin |  | S |  |  |
| Telavancin |  | S |  |  |
| Moxifloxacin | ≤0.25 | S | ≤0.25 | >0.25 |
| Clindamycin | ≤0.25 | S | ≤0.25 | >0.25 |
| Linezolid | ≤4 | S | ≤4 | >4 |
| Levofloxacin | ≤1 | I | ≤0.001 | >1 |
| Oxacillin | 0.25 | * |  |  |
| Gentamicin | ≤1 | * |  |  |
| Gatifloxacin | ≤0.5 | * |  |  |
| Nitrofurantoin | ≤32 | * |  |  |

**Description: S:** sensitive; **I:** intermediary; **R:** drug resistance; ***:** No break point for judgment

**Supplementary Table 2:** Antibiotic Susceptibility of *Methicillin-Resistant* *Staphylococcus aureus* ATCC700699

| **Antibiotic** | **MIC (µg/ml)** | **Result** | **Break P (S)** | **Break P (S)** |
| --- | --- | --- | --- | --- |
| Vancomycin | ≤1 | S | ≤ 2 | > 2 |
| Oritavancin |  | S |  |  |
| Telavancin |  | S |  |  |
| Linezolid | ≤4 | S | ≤4 | >4 |
| Furbenicillin |  | R |  |  |
| Amoxicillin-Flucloxacillin |  | R |  |  |
| Apacillin |  | R |  |  |
| Bacampicillin |  | R |  |  |
| Penicillin | ≥1 | R | ≤0.125 | >0.125 |
| Ampicillin |  | R |  |  |
| Ticarcillin |  | R |  |  |
| Azlocillin |  | R |  |  |
| Mezlocillin |  | R |  |  |
| Doxycycline | ≥16 | R | ≤1 | >2 |
| Azithromycin | ≥8 | R | ≤2 | >2 |
| Levofloxacin | ≥8 | R | ≤0.001 | >1 |
| Teicoplanin | ≤2 | S | ≤2 | >2 |
| Dalbavancin |  | S |  |  |
| Daptomycin | ≤0.5 | S | ≤1 | >1 |
| Tedizolid |  | S |  |  |
| Sulbenicillin |  | R |  |  |
| Flucloxacillin |  | R |  |  |
| Ampicillin-Cloxacillin |  | R |  |  |
| Lemampicillin |  | R |  |  |
| Amoxicillin |  | R |  |  |
| Carbenicillin |  | R |  |  |
| Mecillinam |  | R |  |  |
| Piperacillin |  | R |  |  |
| Tetracycline | ≥16 | R | ≤1 | >2 |
| Erythromycin | ≥8 | R | ≤1 | >2 |
| Trimethoprim-sulfamethoxazole | ≥8/152 | R | ≤2/38 | >4/76 |
| Moxifloxacin | ≥2 | R | ≤0.25 | >0.25 |
| Erythromycin + Lincomycin | ≥4/1 | * |  |  |
| Cefoxitin | ≥16 | * |  |  |
| Rifampicin | ≤0.5 | * | ≤0.06 | >0.06 |
| Clindamycin | 0.5 | R | ≤0.25 | >0.25 |
| Oxacillin | ≥8 | * |  |  |
| Gentamicin | ≥16 | * |  |  |
| Gatifloxacin | ≥4 | * |  |  |
| Nitrofurantoin | ≤32 | * |  |  |

**Description: S:** sensitive; **I:** intermediary; **R:** drug resistance; ***:** No break point for judgment

**Supplementary Table 3:** Antibiotic Susceptibility of *E. coli* ATCC 25922

| **Antibiotic** | **MIC (µg/ml)** | **Result** | **Break P (S)** | **Break P (S)** |
| --- | --- | --- | --- | --- |
| Ampicillin | ≤8 | S | ≤8 | >8 |
| Ceftazidime | ≤1 | S | ≤1 | >4 |
| Cefepime | ≤1 | S | ≤1 | >4 |
| Aztreonam | ≤1 | S | ≤1 | >4 |
| Ampicillin-Sulbactam | ≤8/4 | S | ≤8/4 | >8/4 |
| Levofloxacin | ≤0.12 | S | ≤0.5 | >1 |
| Cefazolin | ≤2 | * |  |  |
| Cefoperazone-Sulbactam | ≤16/8 | * |  |  |
| Amikacin | ≤8 | * |  |  |
| Tigecycline | ≤1 | * |  |  |
| Colistin | ≤1 | * |  |  |
| Piperacillin |  | S |  |  |
| Ceftriaxone | ≤1 | S | ≤1 | >2 |
| Meropenem | ≤1 | S | ≤1 | >8 |
| Piperacillin-Tazobactam | ≤8/4 | S | ≤8/4 | >8/4 |
| Trimethoprim-sulfamethoxazole | ≤2/38 | S | ≤2/38 | >4/76 |
| Cefuroxime | ≤8 | I | ≤0.001 | >8 |
| Cefoxitin | ≤8 | * |  |  |
| Gentamicin | ≤2 | * |  |  |
| Minocycline | ≤4 | * |  |  |
| Nitrofurantoin | ≤32 | * |  |  |

**Description: S:** sensitive; **I:** intermediary; **R:** drug resistance; ***:** No break point for judgment

**Supplementary Table 4:** Antibiotic Susceptibility of *E. coli* (MDR)

| **Antibiotic** | **MIC (µg/ml)** | **Result** | **Break P (S)** | **Break P (S)** |
| --- | --- | --- | --- | --- |
| Ceftriaxone | ≤1 | S | ≤1 | >2 |
| Meropenem | ≤1 | S | ≤2 | >8 |
| Piperacillin-Tazobactam | ≤8/4 | S | ≤8/4 | >8/4 |
| Levofloxacin | 0.25-0.5 | S | ≤0.5 | >1 |
| Ampicillin | ≥32 | R | ≤8 | >8 |
| Trimethoprim-sulfamethoxazole | ≥8/152 | R | ≤2/38 | >4/76 |
| Cefoxitin | ≥32 | * |  |  |
| Gentamicin | ≤2 | * |  |  |
| Minocycline | ≤4 | * |  |  |
| Nitrofurantoin | ≤32 | * |  |  |
| Cefepime | ≤1 | S | ≤1 | >4 |
| Aztreonam | ≤1 | S | ≤1 | >4 |
| Ampicillin-Sulbactam | ≤8/4 | S | ≤8/4 | >8/4 |
| Ceftazidime | 2-4 | I | ≤1 | >4 |
| Cefuroxime | 16 | R | ≤0.001 | >8 |
| Cefazolin | ≥32 | * |  |  |
| Cefoperazone-Sulbactam | ≤16/8 | * |  |  |
| Amikacin | ≤8 | * |  |  |
| Tigecycline | ≤1 | * | ≤0.5 | >0.5 |
| Colistin | ≤1 | * |  |  |

**Description: S:** sensitive; **I:** intermediary; **R:** drug resistance; ***:** No break point for judgment

**Supplementary Table 5:** Antibiotic Susceptibility of *Klebsiella pneumoniae* ATCC1706

| **Antibiotic** | **MIC (µg/ml)** | **Result** | **Break P (S)** | **Break P (S)** |
| --- | --- | --- | --- | --- |
| Ceftazidime | ≤1 | S | ≤1 | >4 |
| Cefepime | ≤1 | S | ≤1 | >4 |
| Aztreonam | ≤1 | S | ≤1 | >4 |
| Trimethoprim-sulfamethoxazole | 4/76 | I | ≤2/38 | >4/76 |
| Cefuroxime | 16 | R | ≤0.001 | >8 |
| Levofloxacin | ≥8 | R | ≤0.5 | >1 |
| Cefoxitin | ≥32 | * |  |  |
| Gentamicin | ≤2 | * |  |  |
| Minocycline | 8 | * |  |  |
| Nitrofurantoin | ≥128 | * |  |  |
| Ceftriaxone | ≤1 | S | ≤1 | >2 |
| Meropenem | ≤1 | S | ≤2 | >8 |
| Piperacillin-Tazobactam | ≤8/4 | S | ≤8/4 | >8/4 |
| Ampicillin | ≥32 | R | ≤8 | >8 |
| Ampicillin-Sulbactam | 16/8 | R | ≤8/4 | >8/4 |
| Cefazolin | ≥32 | * |  |  |
| Cefoperazone-Sulbactam | ≤16/8 | * |  |  |
| Amikacin | ≤8 | * |  |  |
| Tigecycline | 2 | * |  |  |
| Colistin | ≤1 | * |  |  |

**Description: S:** sensitive; **I:** intermediary; **R:** drug resistance; ***:** No break point for judgment

**Supplementary Table 6:** Antibiotic Susceptibility of *Klebsiella pneumoniae* ATCC1705

| **Antibiotic** | **MIC (µg/ml)** | **Result** | **Break P (S)** | **Break P (S)** |
| --- | --- | --- | --- | --- |
| Ampicillin | ≥32 | R | ≤8 | >8 |
| Ceftazidime | ≥32 | R | ≤1 | >4 |
| Cefepime | 16 | R | ≤1 | >4 |
| Aztreonam | ≥16 | R | ≤1 | >4 |
| Ampicillin-Sulbactam | ≥32/16 | R | ≤8/4 | >8/4 |
| Levofloxacin | ≥8 | R | ≤0.5 | >1 |
| Cefoxitin | ≥32 | * |  |  |
| Gentamicin | ≤2 | * |  |  |
| Minocycline | 8 | * |  |  |
| Nitrofurantoin | ≥128 | * |  |  |
| Cefuroxime | ≥32 | R | ≤0.001 | >8 |
| Ceftriaxone | ≥64 | R | ≤1 | >2 |
| Meropenem | ≥16 | R | ≤2 | >8 |
| Piperacillin-Tazobactam | ≥128/4 | R | ≤8/4 | >8/4 |
| Trimethoprim-sulfamethoxazole | ≥8/152 | R | ≤2/38 | >4/76 |
| Cefazolin | ≥32 | * |  |  |
| Cefoperazone-Sulbactam | ≥64/32 | * |  |  |
| Amikacin | 16 | * |  |  |
| Tigecycline | 2 | * |  |  |
| Colistin | ≤1 | * |  |  |

**Description: S:** sensitive; **I:** intermediary; **R:** drug resistance; ***:** No break point for judgment

**
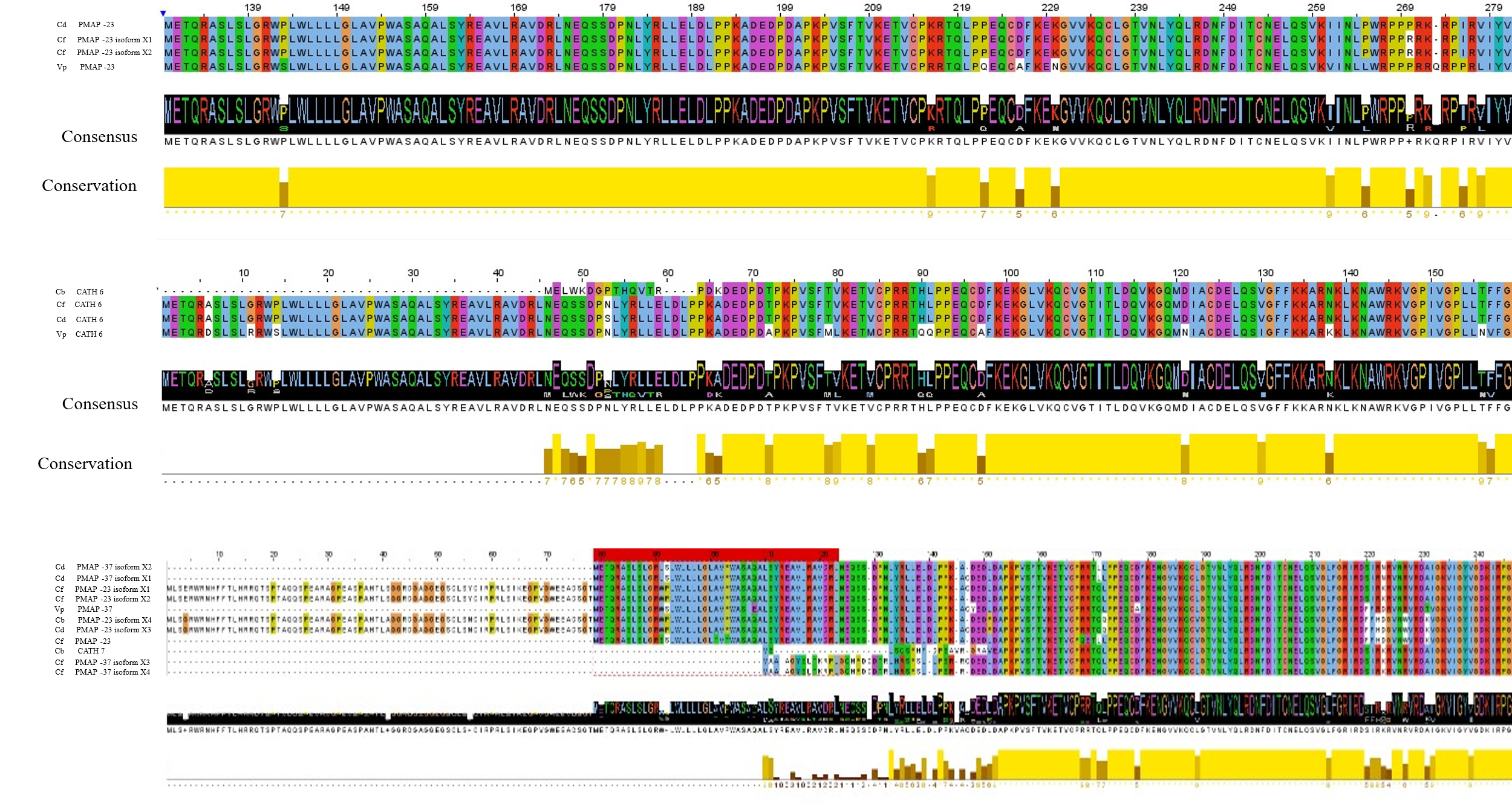
**

**A**

**B**

**C**

**Supplementary Figure 1. Sequence alignment of the identified mature peptides with known cathelicidin peptides of other Camelidae species.**

**A.** The mature CdPMAP-23 is aligned with those from other camel species; the accession numbers of related sequences are: Cf PMAP-23 X1, XP_032314489.1; Cf PMAP-23 X2, XP_032314490.1; Vp PMAP-23, XP_072797299.1. **B.** The mature CdCATH is aligned with those from other camel species; the accession numbers of related sequences are: Cb CATH-6, XP_045364997.1; Cf CATH-6, XP_006173545.1; Cd CATH-6, XP_064352205.1; Vp CATH-6, XP_072796895.1. **C.** The mature CdPG-3 is aligned with those from other camel species; the accession numbers of related sequences are: Cd PMAP-37 X2, XP_064352648.1; Cd PMAP-37 X1, XP_031327002.2; Cf PMAP-23 X1, XP_032314492.1; Cf PMAP-23 X2, XP_032314493.1; Vp PMAP-37, XP_031542475.2; Cb PMAP-23 X4, XP_045361966.1; Cd PMAP-23 X3, XP_045361965.1; Cf PMAP-23, XP_032314504.1; Cb CATH-7, XP_010971471.1; Cf PMAP-37 X3, XP_032314500.1; Cf PMAP-37 X4, XP_032314503.1.

| Peptides | Boman Index (kcal/mol) | Cellular Localization | Total probability of N-in by TMHMM server |
| --- | --- | --- | --- |
| LL-37 (reference peptide) | 2.99 | outside | 0.19240 |
| CdPMAP-23 | 2.02 | outside | 0.29001 |
| CdPG-3 | 3.13 | outside | 0.23791 |
| CdCATH | 0.82 | inside | 0.49030 |

**Supplementary Table 7:** Boman Index and cellular localization of identified peptides

| Peptide | | antiviral activity prediction (AVP) | | | | | Antifungal activity prediction (AFP) | | Hemolysis | |
| --- | --- | --- | --- | --- | --- | --- | --- | --- | --- | --- |
|  | **AVP motif (model)** | | **Alignment model** | **Composition model** | **Physio-chemical model** | **Overall prediction** | **SVM Score** | **Prediction** | **SVM Score** | **Prediction** |
| LL-37 (reference peptide) | Yes | | - | 78.13 % | 72.44 % | Yes | 0.297 | Non-AFP | 0.72 | Non-H |
| CdPMAP-23 | - | | Non-AVP | 41.07 % | 49.90 % | No | 0.009 | Non-AFP | 0.49 | Non-H |
| CdPG-3 | - | | Non-AVP | 40.92 % | 64.13 % | No | 0.363 | Non-AFP | 0.48 | Non-H |
| CdCATH | - | | Non-AVP | 66.08 % | 70.36 % | Yes | 0.178 | Non-AFP | 0.51 | Non-H |
| Inference/ReferenceRange | | - | | | | | <0.5: low probability  >0.5: high probability | | **1** very likely to be hemolytic  **0** very unlikely to be hemolytic | |

**Supplementary Table 8:** Prediction of biological properties including from left to right: antiviral activity prediction (AVP), Antifungal activity prediction (AFP), and Hemolysis.

**Supplementary Table 9:** Prediction of residues composition commonly associated with hemolysis

| Peptide | Sequence | Hydrophobic residues | residues commonly associated with hemolysis | | | | |
| --- | --- | --- | --- | --- | --- | --- | --- |
|  |  |  | **Arginine (R)** | | **Lysine (K)** | | **Tryptophan (W)** |
| LL-37 (reference peptide) | LLGDFFRKSKEKIGKEFKRIVQRIKDFLRNLVPRTES | 27.03% | 13.5% | 16.2% | | 0.0% | |
| CdPMAP-23 | KIINLPWRPPPRKRPIRVIYV | 33.33% | 19.0% | 9.5% | | 4.8% | |
| CdPG-3 | GLFGRIRDSIRNRVNRVRDKVGKVIGYIGDKIRPG | 34.29% | 20.0% | 8.6% | | 0.0% | |
| CdCATH | GFFKKARNKLKNAWRKVGPIVGPLLTFFG | 55.17% | 6.9% | 17.2% | | 3.4% | |

**References**

1. Hussen J. Changes in Cell Vitality, Phenotype, and Function of Dromedary Camel Leukocytes After Whole Blood Exposure to Heat Stress in vitro. Front Vet Sci. 2021 Apr 9;8.
